# A dual-band CPW-fed miniature planar antenna for S-, C-, WiMAX, WLAN, UWB, and X-band applications

**DOI:** 10.1038/s41598-022-11679-7

**Published:** 2022-05-09

**Authors:** Md. Mottahir Alam, Rezaul Azim, Nebras M. Sobahi, Asif Irshad Khan, Mohammad Tariqul Islam

**Affiliations:** 1 Department of Electrical and Computer Engineering, King Abdulaziz University, Jeddah, 21589 Saudi Arabia; 2 Department of Physics, University of Chittagong, Chittagong, 4331 Bangladesh; 3 Computer Science Department, King Abdulaziz University, Jeddah, 21589 Saudi Arabia; 4 Department of Electrical, Electronic & Systems Engineering, Faculty of Engineering and Built Environment, Universiti Kebangsaan Malaysia, 43600 Bangi, Malaysia

**Keywords:** Energy science and technology, Engineering

## Abstract

A miniature planar antenna is a vital component of any portable wireless communication device. The antenna in portable devices should provide wide/multiple operating bands to cover a good number of narrowband services as a multi-band antenna not only reduces the number of antennas but also lessens the system complexity, cost, and device size. To operate over S-, C-, WiMAX, WLAN, UWB, and X-communication bands, in this paper, a dual-band CPW-fed antenna is presented. The anticipated antenna is made up of a vertical bow-tie-shaped patch and two asymmetric ground planes and etched on the same side of the single-sided standard substrate material. To generate two distinct operating bands, an inverted L-shaped parasitic element is inserted within the modified U-shaped coplanar ground plane. The antenna achieved dual operating bands of 3.24–8.29 GHz and 9.12–11.25 GHz in measurement which helps the proposed antenna to cover S-, C-, WiMAX, WLAN, 4G LTE, 5G sub-6 GHz, UWB, and X-communication bands. In the two operating bands, the antenna realized a peak gain of 4.33 dBi, and 4.80 dBi, the maximum radiation efficiency of 86.6%, and 72.6%, and exhibits symmetric radiation patterns. In the operating bands, the antenna also exhibits good time-domain behavior which helps it to transmit the signal with minimum distortion.

## Introduction

As modern wireless communications devices are shifting towards ultra-small sizes, the miniaturization of antennas is indispensable. The design of small wideband/multi-band antennas for handheld communication devices is very tough as it must fulfill broad bandwidth requirements, symmetric radiation pattern, stable gain, compact size, lightweight, and easy fabrication. Recently, there has been increasing attention in searching antennas to cover multiple frequency bands such as S-band (2–4 GHz), WiMAX (3.3–3.8 GHz), 5G mid bands (3.3–3.8 GHz, 4.4–4.9 GHz), C-band (4–8 GHz), WLAN (5.15–5.825 GHz), UWB (3.1–10.6 GHz), X-band (8–12 GHz) due to the numerous operating necessities of communication devices^1–5^. A narrow band microstrip patch antenna can be switched into a multi-band antenna by adding a slit/slot in the patch/ground plane at suitable positions. The size, shape, and placement of this slit/slot play a significant role in the resonances and stopband. If the slot/slit length is either quarter-wavelength or half-wavelength in size at a proper place in the patch/ground plane, it excites the higher modes near the fundamental mode, which helps to achieve multiple operating bands^6^.

Several microstrip line and CPW-fed antennas have already been reported for wideband/multi-band applications. To achieve multi-band operation, they either use slot/s in the patch/ground plane or parasitic slit^7–27^. Unlike microstrip line-fed antennas, CPW-fed antennas occupy nearly all areas surrounding the patch. In a CPW-fed antenna, both the patch and ground plane are designed on one side of the substrate. Due to the existence of the main radiator and the closeness to the ground, CPW feeding is very advantageous for manufacturing antennas. CPW-fed antennas are characteristics with a larger operating band, lower dispersion, and low radiation leakage. Besides, it simplifies construction, allows simple shunt & series surface attachment of additional circuit components, and eliminates the necessity for wraparound and via holes^28^. For example, in Ref. ^9^, a CPW-fed tri-band bow-tie-shaped antenna is reported. By implanting three slots in the bow-tie patch, this design achieved triple operating bands of 2.4 to 2.7 GHz, 3.4 to 3.7 GHz, and 5.2 to 5.8 GHz. However, it has a large size of 100 × 60 mm^2^. In Ref.^10^, a 52 × 64.5 mm^2^ size patch antenna with EBG structure is presented for S-, C-, and X-bands. A pair of II-shaped parasitic slits are used for the excitation of the 2nd and 3rd modes, which helps the antenna to achieve triple bands centered at 3.79 GHz, 7.5 GHz, and 11.8 GHz. For WiMAX and WLAN, a tri-band CPW-fed antenna is reported in Ref. ^11^. This footprint uses inverted L-shaped slots, dipole slots, pairs of slot stubs, and parasitic slots to achieve lower, middle, and upper operating bands. With a dimension of 30 × 65 mm^2^, it achieved triple working bands of 2.375–2.525 GHz, 3.075–3.8 GHz, and 5.0–6.9 GHz. In Ref. ^12^, a tri-band metamaterial-inspired antenna is reported. The designed antenna uses a rectangular complementary split-ring resonator and meandered stripline to achieve multi-band operation. With a 40 mm × 45 mm dimension, it achieved triple working bands of 2.02 to 2.14 GHz, 4.26 to 4.28 GHz, and 5.45 to 5.56 GHz. A radiating patch antenna with an L-shaped slit is reported in Ref. ^13^. A rectangular slot and two C-shaped slots have been inserted near the feedline and defected ground plane to get the desired multi-band operation. In Ref. ^14^, a multi-band slotted antenna is anticipated for LTE, ITU, and X-band communication applications. With a 40 mm × 40 mm dimension, the designed antenna attained triple working bands of 2.20–2.50 GHz, 4.00–4.20 GHz, and 6.70–8.10 GHz. In Ref. ^15^, a triple-band antenna is reported. The studied antenna comprises an L-shaped patch and a ground with an H-shaped slot and can operate over the 2.30–2.75 GHz, 3.19–3.82 GHz, and 5.06–6.15 GHz bands. For WiMAX and WLAN applications, in Ref. ^16^, a wide-slot antenna is presented. It incorporates a fork-shaped patch and a wide slot with a parasitic slit to realize triple operating bands. A CPW-fed antenna is reported in Ref. ^17^. Etching two slots and a split-ring resonator on the ground, the antenna achieved triple operating bands of 2.28–2.56 GHz, 3.29–4.21 GHz, and 5.05–5.91 GHz. In Ref. ^18^, an antenna is reported for WLAN and WiMAX applications. The footprint comprises a four-sided ring, two F-shaped stubs, and a rectangular patch and achieved three working bands of 2.35–2.59 GHz, 3.31–3.93 GHz, and 5.07–6.35 GHz. According to Ref. ^19^, a 30 × 26 mm^2^ antenna has been developed for WLAN and WiMAX bands. The antenna is composed of a pair of metallic loops on the top and a square parasitic ring on the bottom and covers triple bands of 2.33–2.55 GHz, 3.00–3.88 GHz, and 5.15–5.90 GHz. In Ref. ^20^, a tri-band antenna is reported for WiMAX and WLAN. The designed antenna uses a U-shaped patch in the front and a parasitic element on the back to achieve three working bands. A triple-band multi-stubs loaded antenna is reported in Ref. ^21^. The studied design comprises two L-shaped stubs of quarter wavelength and one T-shaped stub and can work over 2.50–2.71 GHz, 3.37–3.63 GHz, and 5.20–5.85 GHz bands. In Ref. ^22^, a dual-band antenna is reported. The slot in this design has a Cantor square Fractal geometry and achieved two distinct bands of 2.35–3.61 GHz and 5.15–6.25 GHz. But it possesses a large physical size of 50 × 50 mm^2^ and does not cover C- and X-bands. A dual-band annular Koch snowflake Fractal antenna is reported in Ref. ^23^. With a 40 mm × 40 mm dimension, the designed antenna realized dual operating bands of 2.24–2.93 GHz and 4.48–5.54 GHz. However, it fails to operate over the WiMAX and X-bands. In Ref. ^24^, a UWB antenna is described for wireless capsule endoscopy. The designed antenna contains a circular patch with a cross-shaped slot and defected ground plane and realized dual operating bands of 5.71–6.28 GHz and 8.13–8.58 GHz. In Ref. ^25^, a tri-band antenna is reported for WLAN and WiMAX applications. With an overall size of 33 × 17 × 1.6 mm^3^, the studied antenna realized triple bands of 2.47–2.77 GHz, 3.3–3.7 GHz, and 5.10–6.62 GHz. However, it does not operate over the X-band. Using a stepped impedance resonator feed line, in Ref. ^28^, a UWB antenna with improved band-edge selectivity is proposed. With an overall size of 24 × 12 mm, the designed antenna achieved a 3.1 to 11 GHz single operating band. However, the design is relatively complex. In Ref.^29^, a UWB antenna is reported for microwave imaging. Several slits are added in the patch and ground plane to enhance the antenna performance, and the designed antenna achieved a single operating band with a bandwidth of 7 GHz. However, the achieved impedance has failed to operate over the S-band, WiMAX, and some of the 5G sub-6 GHz bands. A transmission line-fed UWB antenna with a single notch band for WLAN is presented in Ref.^30^. The H-shaped slots added in the ground plane notch the 4.85–6.17 GHz band, and the antenna failed to cover the WLAN and the upper 5G sub-6 GHz band. Despite the attainment of the dual/triple working band, none of these reported antennas covers X-band (8–12 GHz), even many of them do not cover WiMAX, WLAN, and C-bands. Moreover, some of them possess a large volumetric size and complex footprint. It is, therefore, necessary to introduce an antenna that can work over S-band, WiMAX, 5G mid bands, C-band, WLAN, UWB, and X-bands.

This article introduces a CPW-fed antenna for multi-band communication applications. The presented antenna is comprised of a CPW-fed radiator and two coplanar asymmetrical ground planes. The coplanar ground plane has introduced an inverted L-shaped parasitic element to provide the necessary dual-band operation. The radiation from the ground planes enables the antenna to operate in dual wideband mode with a very compact size. The effect of different parameters on impedance bandwidth is studied using surface current and analyzed in conjunction with supporting parametric analysis. A prototype with optimized parameters is fabricated, and the measured results show that it can work over the 3.24–8.29 GHz and 9.12–11.25 GHz bands.

## Antenna configuration

**Figure 1:**
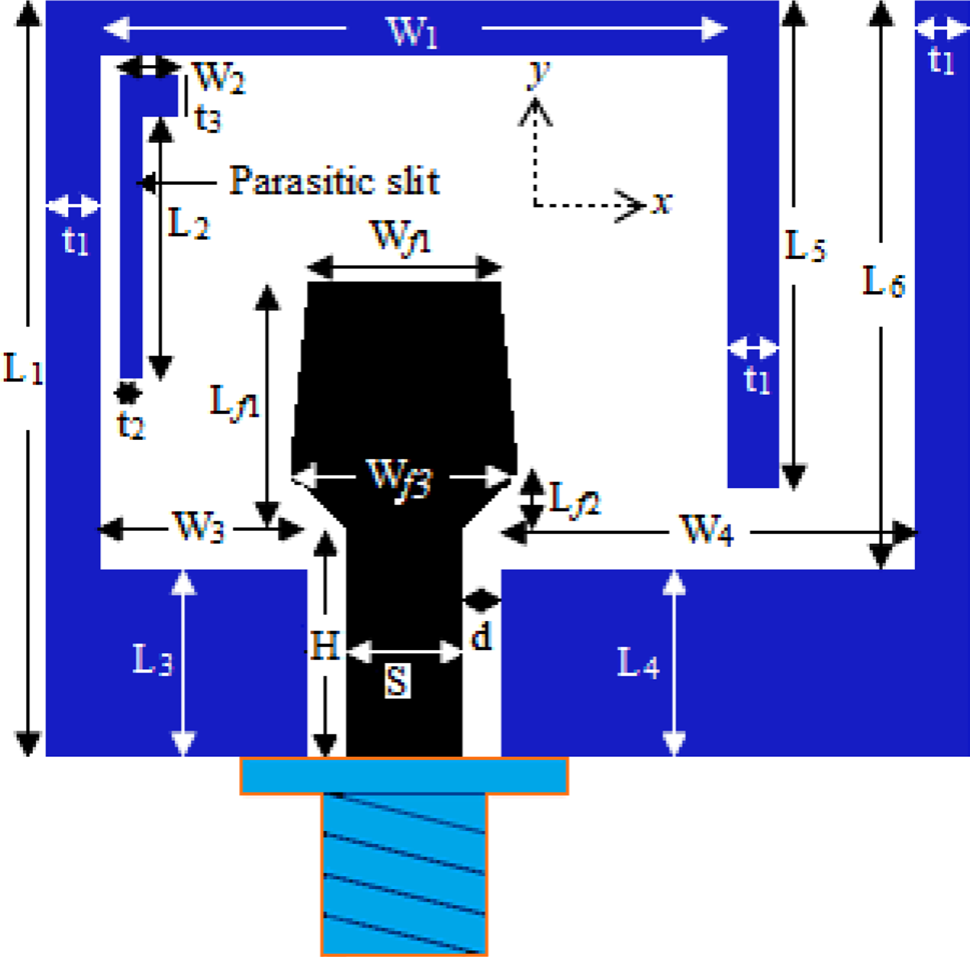
Schematic diagram of the proposed CPW-fed antenna.

The footprint of the studied antenna is depicted in Fig. 1. The anticipated antenna consists of a vertical patch and two asymmetric grounds. The patch and two asymmetric ground planes are designed on the same side of an inexpensive FR4 material with a dielectric consonant (ε_r_) of 4.6, thickness (*h*) 1.6 mm, while the other side is without any materialization of copper. A CPW stripline of width *S* and length *H* is used to feed the antenna. The separation between the patch and ground planes is *d*.

To achieve the wideband matching, the characteristics impedance of the feedline can be calculated by^31^

1 $${Z}_{0}=\frac{30\pi }{\sqrt{{\epsilon }_{e}}}\frac{K\left({k}_{0}^{^{\prime}}\right)}{K({k}_{0})},$$

where ε_e_ is the effective dielectric constant of the substrate and $K\left({k}_{0}\right)$, $K\left({k}_{0}^{^{\prime}}\right)$, $K\left({k}_{1}\right)$, $K\left({k}_{1}^{^{\prime}}\right)$ are the modulus of the complete elliptic integrals and can be calculated using^31^

2 $${\varepsilon }_{e}=1+\frac{({\varepsilon }_{r}-1)}{2}\frac{K({k}_{1})K\left({k}_{0}^{^{\prime}}\right)}{K\left({k}_{1}^{^{\prime}}\right)K({k}_{0})},$$

3 $${k}_{0}=\frac{S}{S+2d},$$

4 $${k}_{0}^{^{\prime}}=\sqrt{1-{k}_{0}^{2},}$$

5 $${k}_{1}=\frac{\text{sinh}(\pi S/4h)}{sinh\left\{\left[\pi (S+2d)\right]/4h\right\}},$$

6 $${k}_{1}^{^{\prime}}=\sqrt{1-{k}_{1}^{2}}.$$

In the above equations, ε and *h* are the relative permittivity and thickness of the substrate. The width *S* and gap *d* of the proposed can be calculated using the above formulas.

The width, *W* of the radiator, which has a slight effect on the resonance, can be obtained using^32^

7 $$W=\frac{c}{{2f}_{r}}\sqrt{\frac{2}{{\varepsilon }_{r}+1}},$$

where c is the speed of light, and ε_r_ is the relative permittivity. As the radiator is on top of the substrate, the effective permittivity of the substrate is^32^

8 $${\varepsilon }_{e}=\frac{({\varepsilon }_{r}+1)}{2}+\frac{({\varepsilon }_{r}-1)}{2}{\left[1+\frac{10h}{W}\right]}^{1/2}.$$

The length, *L* of the radiator, plays a vital role in generating the resonances and can be calculated using^32^

9 $$L=\frac{c}{{2f}_{r}\sqrt{{\varepsilon }_{e}}}-2\Delta L,$$

where $\Delta L$ is the extension of length due to fringing field and can be calculated using^32^

10 $$\Delta L=0.412h\left[\frac{{\varepsilon }_{e}+0.3}{{\varepsilon }_{e}-0.258}\right]\left[\frac{\frac{W}{h}+0.264}{\frac{W}{h}+0.813}\right].$$

The effective length of the radiator is then^32^

11 $${L}_{e}=L+\Delta L.$$

To attain the desired operating band, an inverted L-shaped ground is positioned on the right side of the patch. The L-shaped ground consists of overlapped horizontal and vertical portions with sizes of *W*_4_ × *L*_4_ and *t*_1_ × (*L*_6_ + L_4_), respectively. The area of the overlapped portion is *t*_1_ × *L*_4_. On the left side, one modified inverted U-shaped ground is placed. The modified inverted U-shaped plane comprised of four parts having the area of *W*_3_ × *L*_3_, *t*_1_ × *L*_1_, *W*_1_ × t_1_, and *t*_1_ × *L*_5_. Contrary to the antenna reported in Ref.^33^, in this design, one inverted L-shaped parasitic element with an area of (*t*_2_ × *L*_2_ + *W*_2_ × t_3_) is placed inside the inverted U-shaped ground. This parasitic element and lower end of the patch help the antenna to achieve the higher X-band. The main radiating element of the presented antenna is a vertical bow-tie-shaped patch. The entire length of the patch is *L*_f1_. The length of the tapered section is *L*_f2_, and the length of the upper portion is (*L*_f1_ − *L*_f2_). The lower end of the tapered section is connected to the upper end of the CPW stripline of length *H*. To examine the performances of the presented antenna in terms of achieving the dual operating bands, the IE3D simulator is used for the necessary numerical analysis. The detailed dimension of the anticipated antenna is shown in Fig. 1, and the final dimensional parameters are listed in Table 1.

**Table 1:** Final geometrical parameters of the antenna.

Parameter	Value (mm)	Parameter	Value (mm)	Parameter	Value (mm)
*W*_1_	16.5	*d*	1	*L*_6_	15
*W*_2_	1.5	*L*_1_	20	*L*_f1_	6.5
*W*_3_	5.5	*L*_2_	7	*L*_f2_	1.5
*W*_4_	11	*L*_3_	5	*H*	6
*W*_f1_	5	*L*_4_	5	t_1_	1.5
*S*	3	*L*_5_	13	t_2_	0.5

The equivalent circuit of the studied antenna is a combination of capacitance, inductance, and resistance, and their values are given by Ref.^34^

12 $$C=\frac{{\varepsilon }_{0}{\varepsilon }_{e}WL}{2h}{\text{cos}}^{-2}\left(\frac{\pi S}{2L}\right),$$

13 $$L=\frac{1}{{\omega }^{2}C},$$

14 $$R=\frac{Q}{\omega C},$$

where ω = 2π*f*, and Q is the quality factor having a value of

15 $$Q=\frac{c\sqrt{{\varepsilon }_{e}}}{4{f}_{r}h},$$

with *f*_*r*_ as the resonant frequency. The input impedance of the designed antenna is, therefore

16 $${Z}_{in}=\frac{1}{\frac{1}{R}+\frac{1}{j\omega L}+j\omega C}.$$

Therefore, the reflection coefficient and return loss values can be evaluated using^31^

17 $$\Gamma =\frac{{Z}_{0}-{Z}_{in}}{{Z}_{0}+{Z}_{in}},$$

18 $${Return loss}_{dB}=20log\left|\Gamma \right|$$

**Figure 2:**
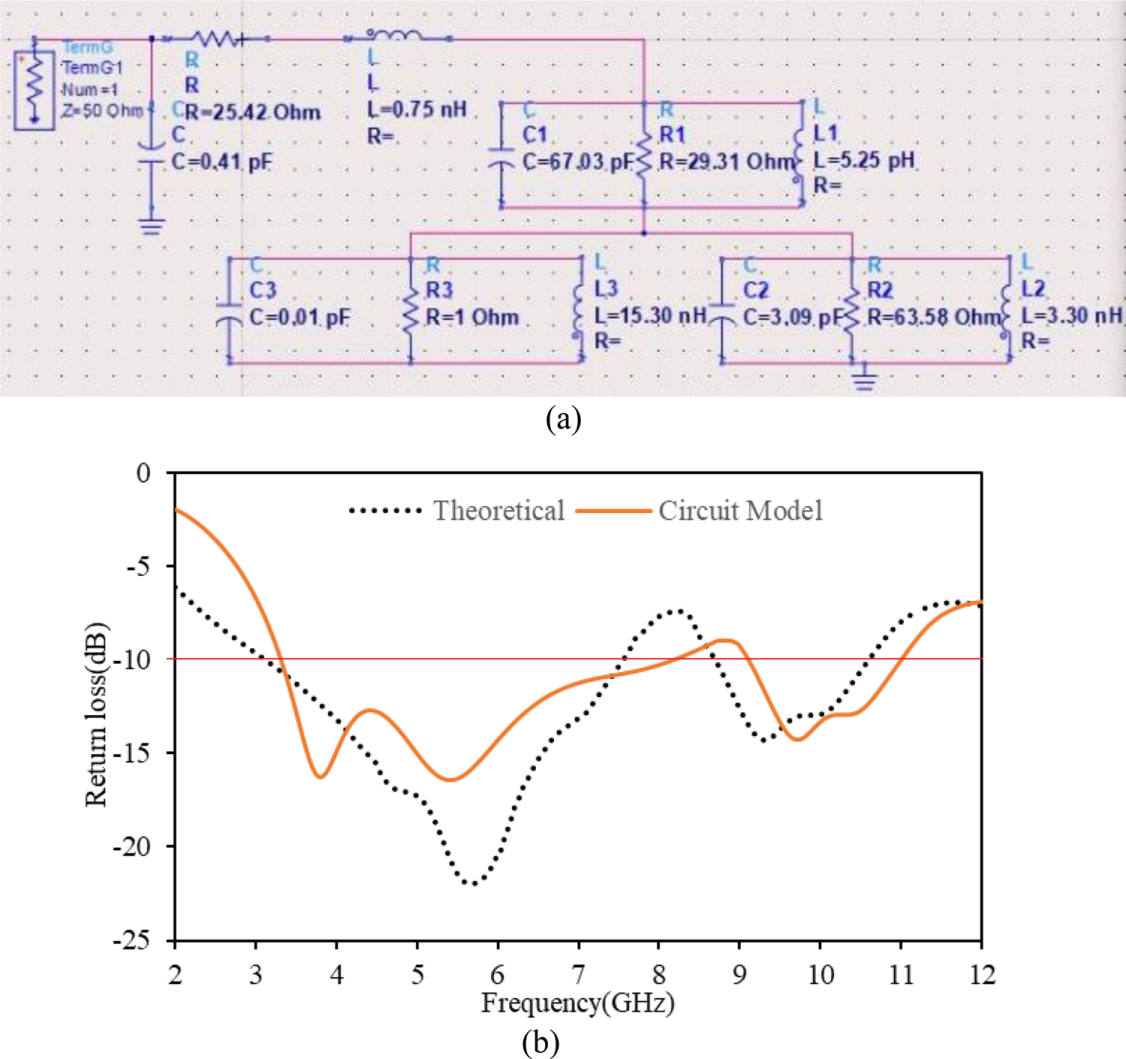
(**a**) Lumped equivalent circuit model and (**b**) theoretical & circuit model return loss curves of the antenna.

After extracting and calculating the parameters from the simulation, a lumped equivalent circuit model of the anticipated antenna is constructed and is portrayed in Fig. 2a. Additionally, this layout is verified by circuit theory analysis using advanced design system (ADS). The theoretical and lumped circuit model return loss curves are depicted in Fig. 2b. Despite the slight discrepancy between theoretical and lumped model return losses, it is evident that the designed antenna realized dual operating bands and is suitable for S-, C-, WiMAX, WLAN, UWB, and X-band applications.

## Design investigation and analysis

### Design evolution

**Figure 3:**
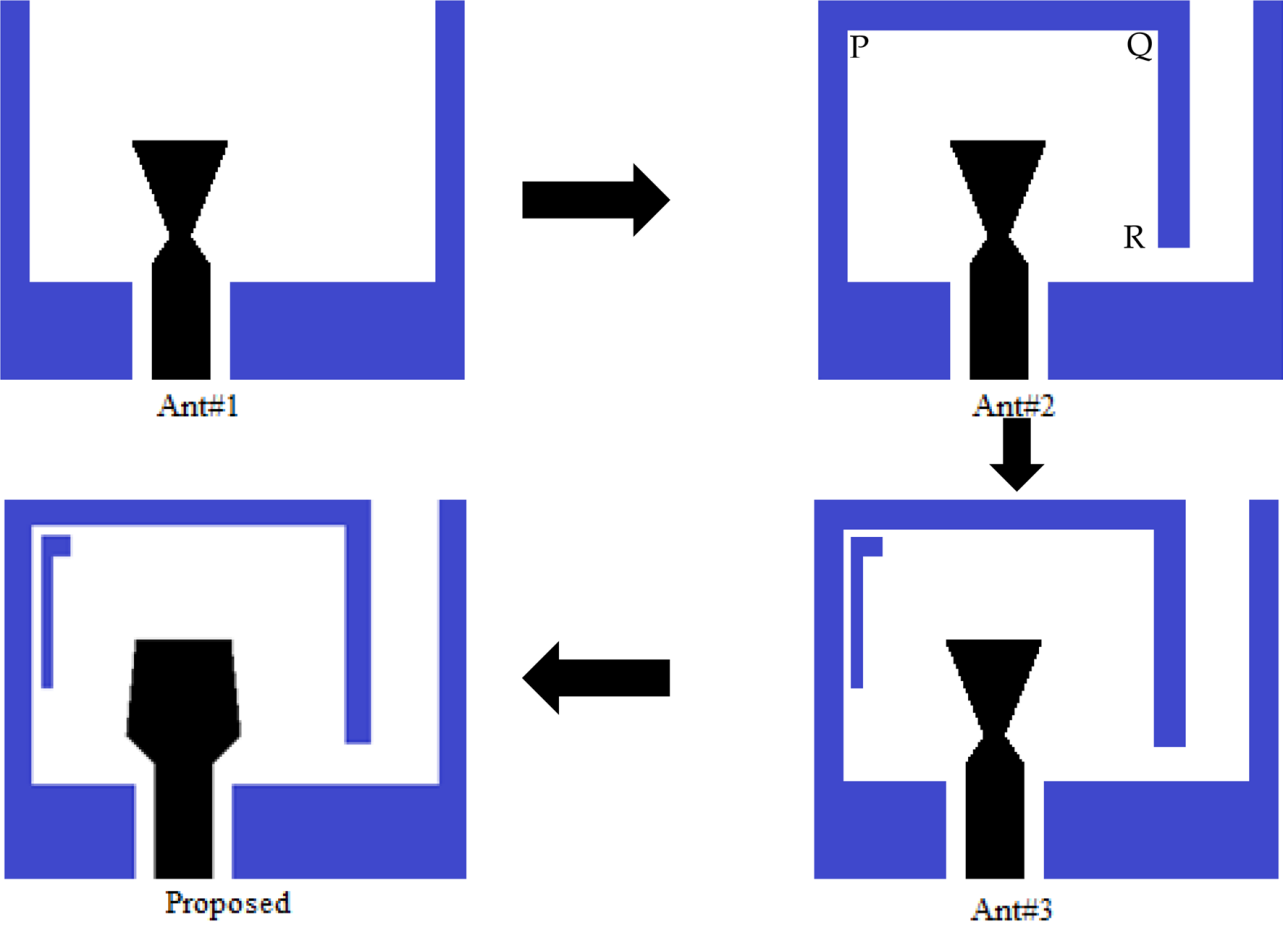
Design evolution of the proposed antenna.

**Figure 4:**
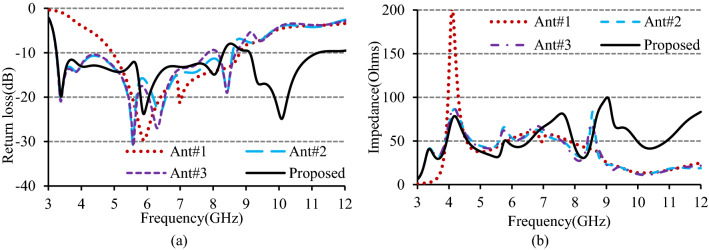
(**a**) Return loss curves, and (**b**) input impedances for the different evolution stages.

To know the operating process and effect of different parts, Fig. 3 portrays the development of the expected antenna. Figure 4 illustrates the return losses and input impedances for various phases of evolution. In the development procedure, the initial design Ant#1 includes one L-shaped and one inverted L-shaped ground plane and a CPW-stripline-fed bow-tie-shaped patch. To form Ant#2, a section, PQR consisting of two rectangular bars of area *W*_1_ × *t*_1_ and *t*_1_ × *L*_5_ is incorporated at the upper part of the L-shaped ground plane, which in-turns convert into a modified inverted U-shaped ground. In Ant#3, one inverted L-shaped parasitic element of area (*t*_2_ × *L*_2_ + *W*_2_ × *t*_3_) is placed inside the U-shaped ground. Finally, the anticipated antenna is formed by adding two triangular sections to the patch's left and right lower end, as depicted in Fig. 3.

The return loss curve displayed in Fig. 4a shows that Ant#1 can operate over a 4.94–8.81 GHz frequency band, where the impedance is very much near to 50Ω. The addition of conducting section PQR to the L-shaped ground of Ant#1 increases the inductive effect of the antenna. This enhanced inductive effect due to a more protracted current path introduced three extra resonances at around 3.39 GHz, 3.81 GHz, and 8.37 GHz and helped Ant#2 in exhibiting a working band of 3.24–8.60 GHz. As shown in Fig. 4b, this band’s input impedance is better than Ant#1. When the parasitic element is positioned inside the modified U-shaped ground to form Ant#3 (Fig. 3), a stopband ranging from 7.84 to 8.14 GHz is created, and two separate working bands of 3.24–7.84 GHz and 8.14–8.69 GHz are exhibited as shown in Fig. 4a. This is due to the extra capacitive effect between the parasitic element and inverted U-shaped ground. When two triangular sections are added to the left and right lower end of the patch to look hexagonal, the inductive and the capacitive effects are increased further. The increased inductive and capacitive effects widen the stopband and move the higher resonance near the upper band, as portrayed in Fig. 4a. The final anticipated antenna exhibits two distinct operating bands of 3.248–8.31 GHz and 9.11–11.19 GHz. In the two bands, the impedance matching is much better and nearly equal to 50Ω.

### Computation of equate-length of the current path

**Figure 5:**
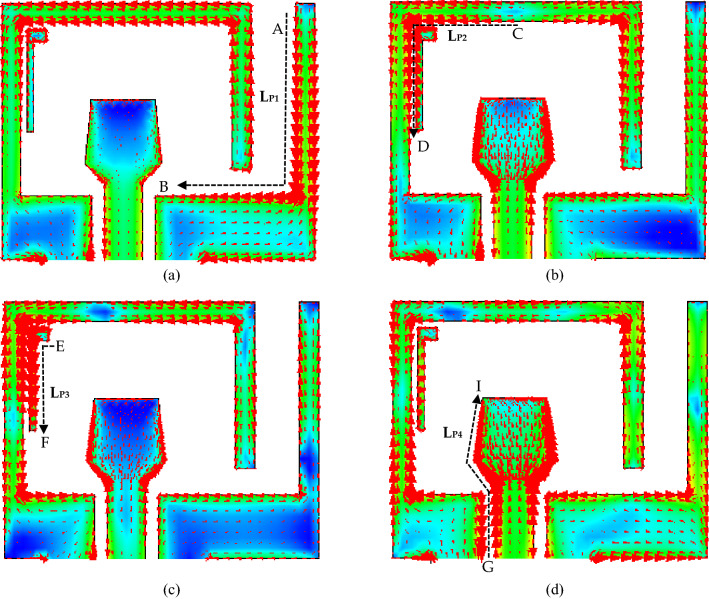
Current distributions at (**a**) 3.375, (**b**) 5.90, (**c**) 7.98, and (**d**) 10.1 GHz.

The surface current distribution on the antenna at four resonance frequencies of 3.375 GHz, 5.90 GHz, 7.98 GHz, and 10.1 GHz are presented in Fig. 5. In the figure, *L*_P1_, *L*_P2_, *L*_P3_, and *L*_P4_ displayed four current streamlines. In Fig. 5, the arrow signs show the current direction, the red color signifies the peak current density, and the blue color indicates a null. The effective length of the current path is evaluated analytically and produced directly from the CST simulator. To calculate the current streamline lengths, the numerical values of the geometrical parameters of Table 1 are used. At the fundamental resonant mode of 3.375 GHz, as portrayed in Fig. 5a, the current around the inner edge of the inverted L-shaped ground is significantly larger than other portions of the antenna. The first streamline length *L*_P1_ corresponding to the first resonance frequency is

19 $${L}_{P1}=AB={L}_{6}+{W}_{4}=26 \text{ mm}.$$

This current path length is slightly longer than the corresponding quarter wavelength. As shown in Fig. 5b, at the first excited mode of 5.9 GHz, maximum currents are concerted in section CD of the inverted U-shaped ground. The second streamline length *L*_P2_ corresponding to this resonance frequency is

20 $${L}_{P2}=CD=\frac{{W}_{1}}{2}+\frac{{L}_{2}}{2}=11.75 \text{ mm},$$

which is also one-quarter of the wavelength. At 7.98 GHz, as in Fig. 5c, the second excited resonance is primarily due to the strongest current flow in the parasitic element. Here the third streamline length *L*_P3_ is

21 $${L}_{P3}=EF={W}_{2}+{L}_{2}=8.5\text{ mm},$$

which is again a quarter wavelength of the corresponding wavelength. As shown in Fig. 5d, at *f* = 10.1 GHz, the fourth resonance is essentially owing to the monopole behavior of the vertical radiator. The streamline length *L*_P4_ for this resonance is

22 $${L}_{P4}=GI=H+\sqrt{{L}_{f2}^{2}+{\left(\frac{{W}_{f3}-S}{2}\right)}^{2}}+\sqrt{{\left({L}_{f1}-{L}_{f2}\right)}^{2}+{\left(\frac{{W}_{f3}}{2}\right)}^{2}}=13.95\text{ mm},$$

and is a bit shorter than half of the wavelength. From the current distribution, it is clear that three dominant current streamlines have been observed in the first working band of 3.248 to 8.31 GHz, and one dominant streamline of current has been spotted in the second working band, which generates the fourth resonant mode. It is also worthy of revealing that the current streamline is nearly one-quarter/half of the wavelength at corresponding frequencies in the resonances. The small shifting from the quarter/half wavelength is primarily owing to the additional loading effect from the non-resonating parts of the antenna.

### Parametric study

**Figure 6:**
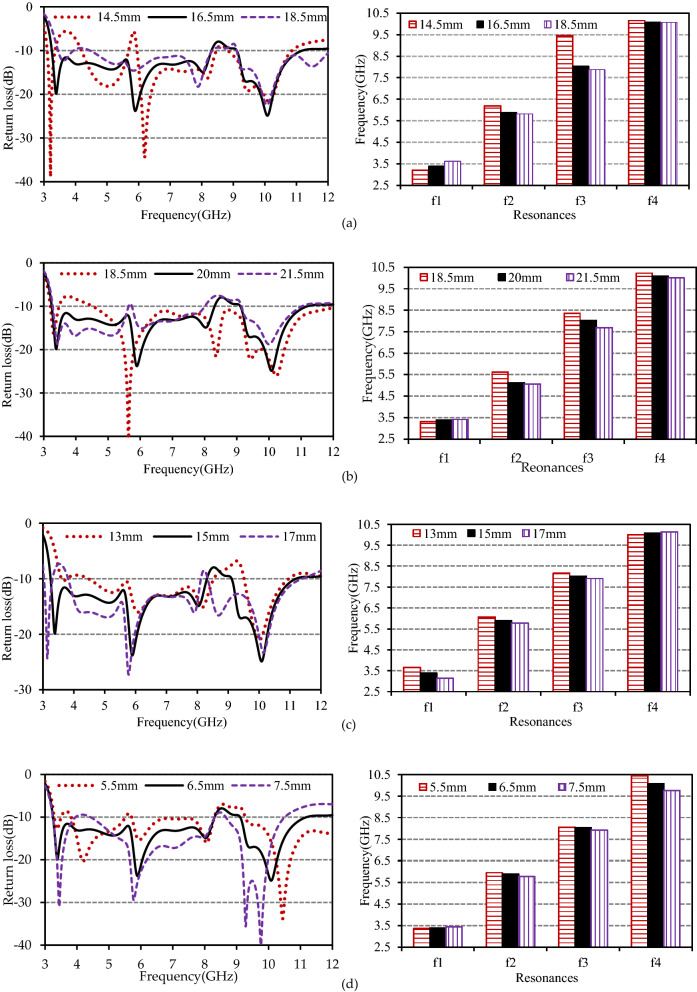

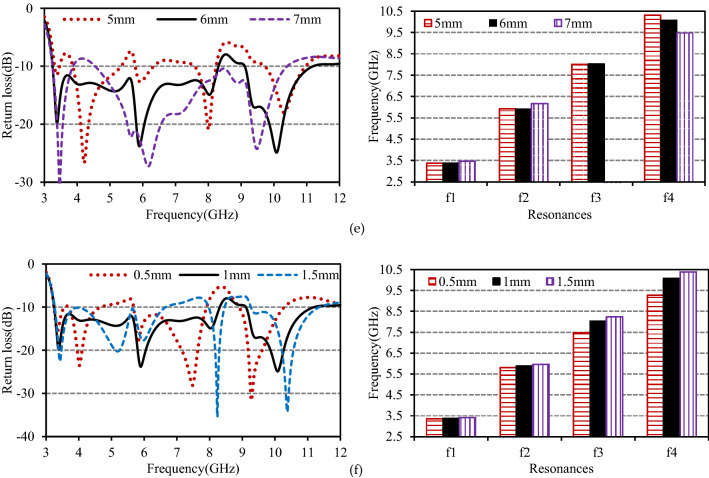
(**a**) Effect of *W*_1_ on return losses and resonance frequencies, (**b**) effect of *L*_1_ on return losses and resonance frequencies, (**c**) effect of *L*_6_ on return losses and resonance frequencies, (**d**) effect of *L*_f1_ on return losses and resonance frequencies, (**e**) effect of *H* on return losses and resonance frequencies, and (**f**) effect of *d* on return losses and resonance frequencies.

A parametric study was undertaken to investigate the impact of various parameters on antenna performance, which enables a more detailed optimization process. To inspect the sensitivity of *W*_1_ on antenna performances and resonance frequencies, the change of return loss and resonances are portrayed in Fig. 6a. As predicted, with the increment of *W*_1,_ the first resonance moves towards a higher band while the second and third resonances shift downward, and the fourth resonance remains unaltered. The plot shows that the second and third resonances decrease with increasing *W*_1_. This is also justified by the mathematical expression of *L*_P2_ mentioned in Eq. (20). Figure 6b portrayed the effect of changing *L*_1_ on the resonances and antenna performances. It is seen that the length *L*_1_ has a minimal impact on the first resonance (*f*_1_) and fourth resonance (*f*_4_) while it influences the second (*f*_2_) and third (*f*_3_) resonances. Both the second and third resonance frequencies tend to decrease as *L*_1_ is increased from 18.5 to 21.5 mm. This may be a way to generate a stop band at around 8.22 GHz. The equation for the first current path, *L*_P1_, leads us to the effect of changing *L*_6,_ which is verified by the parametric study portrayed in Fig. 6c. As the value of *L*_6_ changes from 13 to 17 mm, the first resonance frequency decreases from 3.66 to 3.14 GHz. The evolution of *L*_6_ also has some additional effects on the second and third resonances as well as the stopband at around 8.37 GHz. The current streamline length of the fourth resonance, *L*_P4_, demonstrated a strong dependence on *L*_f1_ and *H* portrayed in Fig. 6d,e, respectively, keeping all other parameters unaltered. The fourth resonance frequency changes from 10.31 to 9.76 GHz when the value of *L*_f1_ increases from 5.5 to 7.5 mm. On the other hand, the fourth resonance point decreases from 10.31 to 9.47 GHz as the value of *H* changes from 5 to 7 mm. However, the change of *L*_f1_ and *H* affects other resonances and stopbands. In addition to the above parameters, the coupling gap between the feedline and coplanar ground planes, *d,* dramatically influences the two operating bands, especially on the third and fourth resonances. The effect of *d* on the return loss and resonances is depicted in Fig. 6f. The plot shows that the third and fourth resonance frequencies sharply increased when the value of *d* increased from 0.5 to 1.5 mm. The first and second resonance points also slightly rise with *d*. Moreover, the bandwidth of the stopband at around 8.49 GHz is increased with decreasing coupling gap between the radiator and coplanar ground plane.

## Results and discussion

### Frequency-domain analysis

**Figure 7:**
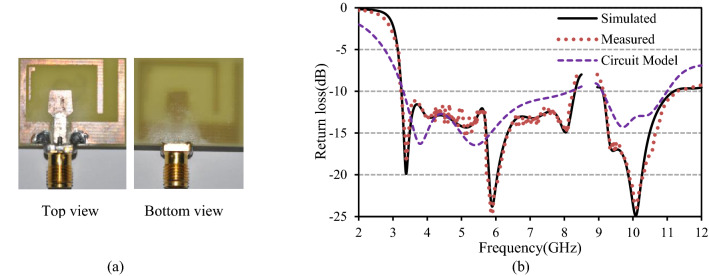
(**a**) Prototype of the designed antenna, (**b**) comparison of return losses versus frequency.

The anticipated antenna has been fabricated for experimental verification, and its performance is measured using the N5227A network analyzer from Keysight Technologies. The fabricated antenna is displayed in Fig. 7a, and its simulated, measured, and circuit model return loss curves are shown in Fig. 7b. A good agreement between the results has been observed. For RL ≥  − 10 dB, the measured results portrayed that the anticipated antenna produces dual working bands with a bandwidth of 5.05 GHz (3.24–8.29 GHz, 87.6%) and 2.13 GHz (9.12–11.25 GHz, 20.9%). The slight disagreement may be attributed to the prototyping and measurement limitations.

**Figure 8:**
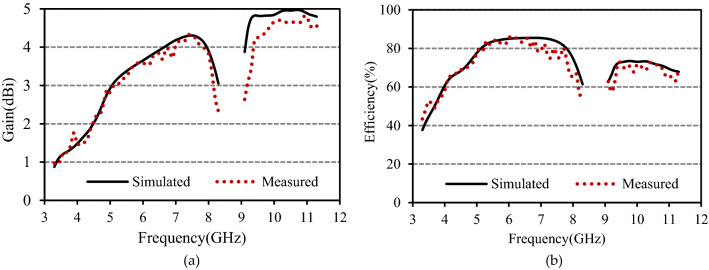
(**a**) Gain and (**b**) efficiency of the presented antenna.

The radiation characteristics of the antenna have been measured in MVG’s near-field antenna measurement system, StarLab. Figure 8a portrayed the gain of the anticipated antenna. The plot demonstrates that in the first and second bands, the average measured gains are 2.98 dBi and 4.37 dBi, with the maximum values of 4.33 dBi and 4.8 dBi. The studied antenna’s radiation efficiency is displayed in Fig. 8b. A good match between the simulated and measured efficiency has been observed. The plot shows that the antenna attained an average efficiency of 72.4% and 68.4% in the lower and higher band. The maximum efficiency at lower and higher bands is 86.6% and 72.6%, respectively.

**Figure 9:**
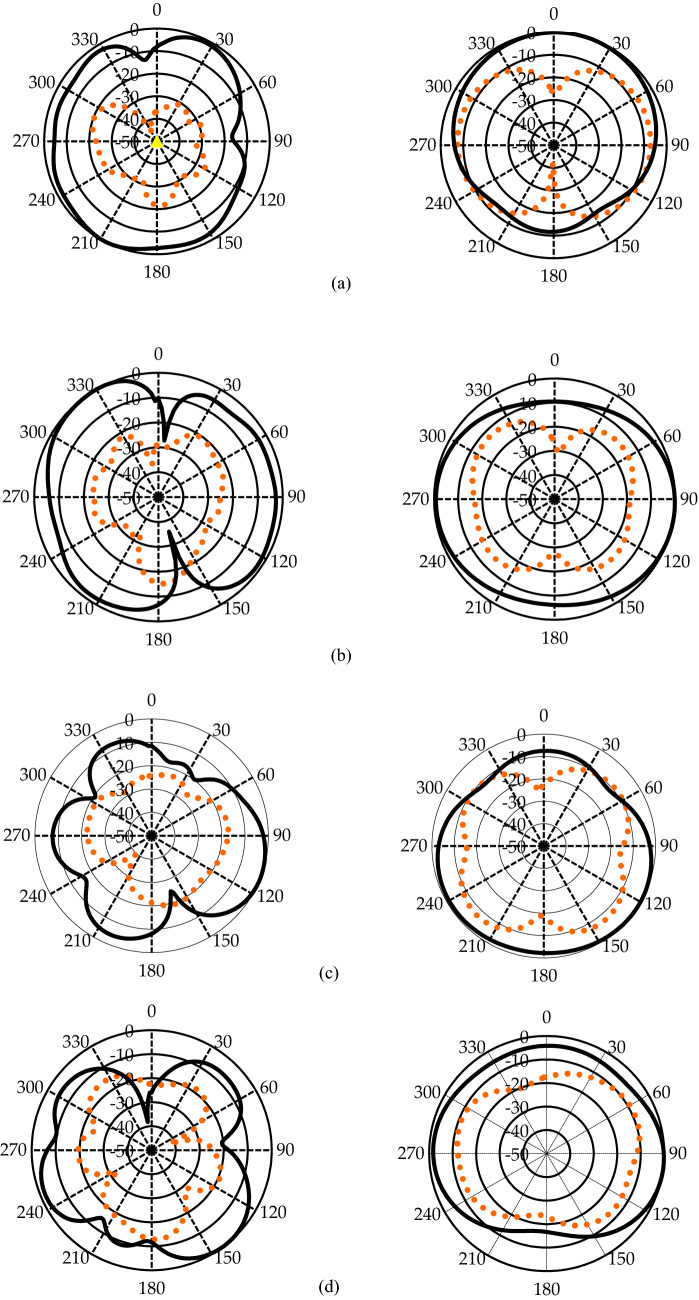
Measured radiation pattern at (**a**) 3.375 GHz, (**b**) 5.90 GHz, (**c**) 7.98 GHz, and (**d**) 10.1 GHz.

The anticipated antenna’s measured *E*(yz) and *H*(xz)-field patterns at four resonance frequencies of 3.375 GHz, 5.90 GHz, 7.98 GHz, and 10.1 GHz are portrayed in Fig. 9. It can be observed that the anticipated antenna demonstrates a nearly omnidirectional radiation pattern. Despite an asymmetrical structure, the antenna exhibits symmetrical radiation patterns over the two bands with no back lobes. The primary benefit of the antenna’s symmetric radiating patterns is that the highest power is focused in the broadside direction that is unaffected by frequency changes. Therefore, the antenna exhibits broad half-power beamwidth in both *E*- and *H*-planes. At the low frequency of 3.375 GHz, the cross-polarization level of the *H*-plane pattern is more significant, which may be owing to the degeneration of the small antennas^35–37^. Some nulls have also been observed, especially in the *E*-plane, mainly due to the agitation of the higher-order current modes. Despite these nulls, the radiation patterns of the studied antenna are still symmetrical, which is a primary requisite for WiMAX, WLAN, UWB, etc., applications.

### Time-domain analysis

As a UWB antenna directly transmits narrow pulses, it is essential to inspect its time-domain behavior. The easiest method to test the time-domain behavior is to find the fidelity factor (FF). The FF measures the degrees of similarity between the transmitted and received signals. The FF can be calculated using^38^

23 $$\text{FF}=\text{Max}\left[\frac{{\int }_{-\infty }^{+\infty }{T}_{x}\left(t\right){R}_{x}\left(t+T\right)dT}{{\int }_{-\infty }^{+\infty }{\left|{T}_{x}\left(t\right)\right|}^{2}dt{\int }_{-\infty }^{+\infty }{\left|{R}_{x}\left(t\right)\right|}^{2}dt}\right],$$

where T_x_(t) and R_x_(t) are respectively the transmitted and received signals.

**Figure 10:**
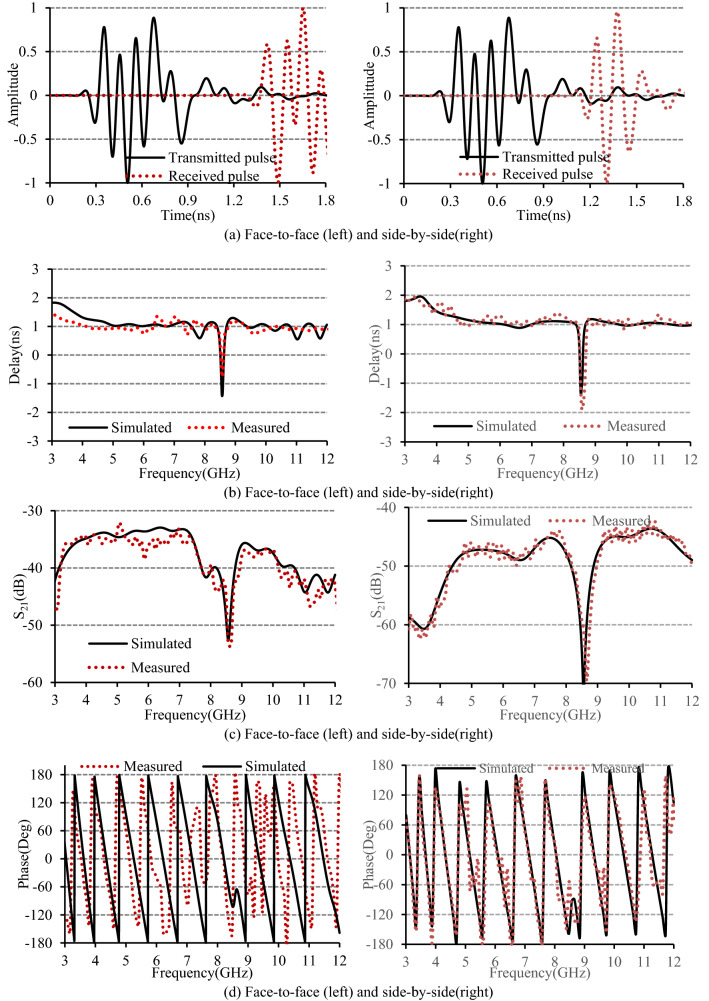
(**a**) Transmitted and received signal, (**b**) group delay, (**c**) transfer function (S_21_), and (**d**) phase of S_21_.

A pair of transmitting and receiving antennas are positioned at 240 mm apart to calculate the FF using the CST simulator. A Gaussian pulse is selected for transmission, covering the entire UWB spectral mask. A number of virtual probes are positioned every 10° from 0° to 90°. Figure 10a displayed the normalized transmitted and received pulses. It is revealed that the FF is around 89.6% in face-to-face position, which has a value of 85.5% in side-by-side placement. These higher values of the FF indicate that the designed antenna can receive the input signal without any distortion. The group delay that quantifies the dispersion of the transmitted electromagnetic signal is defined as the negative derivative of the phase to frequency^38^. Contrary to the narrowband counterpart, the group delay of an ultra-wideband antenna may vary significantly. The group delay should be small or flat in the working band for distortion-free transmission. The group delay is measured by exciting two identical antennas kept in the far-field region, maintaining a distance of 240 mm between them. The group delay of the studied antenna at the 3–12 GHz band is depicted in Fig. 10b. It can be seen from the plot that except at the stopband of 8.3–9.11 GHz, the group delay is almost flat over the two operating bands. The average measured group delay in face-to-face alignment is 1.02 ns and 0.92 ns in the first and second bands. The first and second bands’ measured group delays are 1.26 ns and 1.07 ns in a side-by-side alignment. These slight variations ensure the phase linearity of the transmitted signal. Figure 10c demonstrates the simulated and measured transfer function for a pair of antennas in face-to-face and side-by-side placement. Relatively flat values of the transfer function have been seen in the functional bands with little variations in the stopband. The transfer function’s measured and simulated phase distribution for both placements, shown in Fig. 10d, is linear except at the stopband. The smooth simulated result can be attributed to the distortion-free simulation setup.

A comparison of the presented antenna with recently proposed dual/multi-band antennas is summarized in Table 2. As the studied antenna is smaller than existing antennas and can operate in two different frequency bands, it is ideal for use in the S-, C-, WiMAX, WLAN, 4G LTE, 5G sub-6 GHz, UWB, and X-communication bands. Although some antennas' working bands and bandwidth are higher than those of the proposed antenna, the studied design offers the advantages of a compact footprint, a smaller physical dimension, and ease of commercial production for small-volume multi-band antenna applications.

**Table 2:** Comparison of the proposed antenna with recently reported multi-band antennas.

References	Dimension (mm^2^)	Operating bands (GHz)	Peak gain (dBi)	Substrate ε_r_	Design complexity
Ref.^9^	100 × 60 = 6000	2.17–2.72	3.75	FR4 4.2	Moderate
		3.34–3.66	3.56		
		4.85–5.77	3.93		
Ref.^11^	30 × 65 = 1950	2.375–2.525	1.30	FR4 4.3	Moderate
		3.075–3.80	5.20		
		5.00–6.90	3.10		
Ref.^12^	40 × 45 = 1800	2.02–2.14	1.87	FR4 4.4	Complex
		4.26–4.28	2.90		
		5.45–5.56	4.13		
Ref.^13^	33 × 50.9 = 1680	2.80–3.00	2.32	FR4 4.4	Moderate
		3.30–3.50	1.21		
		3.80–8.00	− 6.00		
Ref.^14^	40 × 40 = 1600	2.20–2.50	NR	FR4 4.4	Complex
		4.00–4.20			
		6.70–8.10			
Ref.^15^	37 × 40 = 1480	2.30–2.75	2.50	FR4 4.4	Complex
		3.19–3.82	0.83		
		5.06–6.15	2.02		
Ref.^16^	28 × 32 = 896	2.29–2.88	4.10	FR4 4.4	Moderate
		3.26–3.88	4.33		
		4.17–6.07	2.70		
Ref.^17^	23 × 38 = 874	2.28–2.56	1.72	FR4 4.4	Moderate
		3.29–4.21	2.66		
		5.05–5.91	3.10		
Ref.^18^	23 × 36 = 828	2.35–2.59	2.93	FR4 4.4	Simple
		3.31–3.93	2.25		
		5.07–6.35	3.04		
Ref.^19^	26 × 30 = 780	2.33–2.55	1.24	NR 2.65	Moderate
		3.00–3.88	2.92		
		5.15–5.90	2.87		
Ref.^20^	20 × 38.5 = 770	2.10–2.49	2.95	FR4 4.4	Complex
		3.22–4.30	3.30		
		4.89–6.12	3.05		
Ref.^21^	24 × 30 = 720	2.50–2.71	1.93	Rogers 2.2	Simple
		3.37–3.63	1.89		
		5.20–5.85	1.94		
Ref.^22^	50 × 50 = 2500	2.35–3.61	2.8–3.5	FR4 4.4	Moderate
		5.15–6.25	3.7–4.3		
Ref.^23^	40 × 40 = 1600	2.24–2.93	2.8–3.3	Rogers 4.5	Complex
		4.48–5.54	3.2–5.7		
Ref.^25^	33 × 17 = 561	2.47–2.77	2–3.8	FR4 NR	Moderate
		3.30–3.70			
		5.10–6.62			
Ref.^39^	18 × 34.5 = 612	2.28–2.57	≈ 1.40	FR4 4.4	Simple
		5.00–6.27	≈ 2.70		
		7.11–7.96	≈ 3.35		
Ref.^40^	50 × 30 = 1500	1.42–2.08	2.10	FR4 4.4	Moderate
		3.49–4.13	1.75		
		5.23–7.53	2.50		
		8.00–9.87	5.50		
		10.7–20.0	6.50		
Ref.^41^	40 × 49 = 1960	Centred at 3.54 and 6.72 GHz	4.78	FR4 2.65	Moderate
			4.65		
Ref.^42^	24 × 32 = 768	3.1–4.5	3.80	RT/Duroid 5880 2.2	Complex
		10.3–11.7	3.60		
Ref.^43^	58 × 40 = 2320	Centred at 2.5 and 4.2 GHz	3.20	FR4 4.4	Moderate
			5.20		
Ref.^44^	29 × 30 = 870	3.00–3.25	NR	Rogers 2.2	Moderate
		3.60–4.40			
		4.80–5.10			
		5.40–5.75			
		5.95–7.50			
		8.75–9.00			
Ref.^45^	96 × 100 = 9600	1–30	2.2–11	Rogers 3.38	Moderate
Proposed	24.5 × 20 = 490	3.24–8.29	4.33	FR4 4.6	Simple
		9.12–11.25	4.80		

## Conclusions

Currently, many portable communication devices require an antenna that is small, planar, and operable over multiple bands to cover a good number of communication services. This paper proposes a dual-band CPW-fed antenna with two asymmetrical radiating coplanar ground planes. Including an inverted L-shaped parasitic slit helps the designed antenna generate two distinct operating bands. The fabricated antenna shows dual operating bands of 3.24–8.29 GHz (5.05 GHz) and 9.12–11.25 GHz (2.13 GHz). The antenna achieved the highest gain of 4.33 dBi and 4.8 dBi and the highest efficiency of 86.6% and 72.6% in the two working bands. The antenna also exhibits stable radiation patterns and excellent time-domain characteristics in the two operating bands, making it suitable for handheld communication devices. The prime advantage of the studied antenna is that it covers different service bands, including S-, C-, WiMAX, WLAN, UWB, and X-band communication systems, with very compact size (24.5 × 20 mm^2^) and a uniplanar profile.
